# Within-Athlete Associations Between WHOOP Activity Strain, Coach-Prescribed Training Objectives, and Session-RPE in an Elite Junior Swimmer: A Longitudinal Single-Case Study

**DOI:** 10.3390/jfmk11030369

**Published:** 2026-09-11

**Authors:** Árpád Petrov, István Barthalos, Daniel Arany, Katalin Nagyváradi, Iván Petrov, Zoltán Alföldi, Ferenc Ihász

**Affiliations:** 1 Doctoral School of Regional and Economic Sciences, Széchenyi István University, 9026 Győr, Hungary; petrov.arpad@gmail.com; 2 Department of Health Promotion and Exercise Sciences, Széchenyi István University, 9026 Győr, Hungary; barthalos.istvan@sze.hu (I.B.); alfoldi.zoltan@sze.hu (Z.A.); ihasz.ferenc@ga.sze.hu (F.I.); 3 Doctoral School of Education, Faculty of Pedagogy and Psychology, Eötvös Loránd University, 1075 Budapest, Hungary; 4 Institute of Sport Sciences, Faculty of Education and Psychology, Eötvös Loránd University, 9700 Szombathely, Hungary; nagyvaradi.katalin@ppk.elte.hu; 5 Doctoral School of Health Sciences, Faculty of Health Sciences, University of Pécs, 7621 Pécs, Hungary; petrovswim@gmail.com; 6 Heart and Vascular Centre, Semmelweis University, 1085 Budapest, Hungary; 7 Department of Sports Medicine, Semmelweis University, 1085 Budapest, Hungary

**Keywords:** elite junior swimmer, wearable technology, WHOOP activity strain, session-RPE, training objective, longitudinal single-case study, generalized estimating equations

## Abstract

**Background and Objectives:** Wearable technologies increasingly monitor internal training load in high-performance sport, but their alignment with coach-prescribed physiological objectives remains unclear. This study examined within-athlete associations between WHOOP Activity Strain, coach-prescribed objectives, athlete-perceived load, and completed swimming exposure in one elite junior swimmer. **Methods:** An exploratory longitudinal single-case secondary analysis was conducted using routinely collected training and athlete-monitoring data from 4 January to 3 July 2026. WHOOP Activity Strain and Session-RPE were synchronized by session, and generalized estimating equations assessed differences across prescribed objectives and variable associations. **Results:** Of 161 sessions with exact-plan cards, 134 were classified and 128 had strict WHOOP matches. Conventional robust-covariance GEE suggested an objective-related difference (exchangeable *p* = 0.013; AR(1) *p* = 0.014), but this was not statistically detectable with the bias-reduced covariance estimator (*p* = 0.080 and *p* = 0.075, respectively) and was not reproduced using the legacy classification. WHOOP Activity Strain was consistently associated with Session-RPE Load, Session-RPE, and actual swimming distance. **Conclusions:** Within this athlete, WHOOP Activity Strain was associated with perceived load and completed swimming distance. Objective-related differences were post hoc and sensitive to the classification source and covariance estimator; they should therefore be considered exploratory and hypothesis-generating.

## 1. Introduction

Physiological responses elicited by exercise contribute to subsequent training adaptation; however, the magnitude and direction of performance change also depend on training history, recovery, sleep, nutrition, psychological state, and growth and maturation. Training-load monitoring is therefore used to place the completed work and the athlete’s response into context, rather than to reduce adaptation to a single score [1,2,3,4,5,6].

Wearable systems have expanded longitudinal monitoring in routine training. These systems combine signals such as heart rate, heart rate variability (HRV), sleep characteristics, movement, and algorithm-derived readiness or load scores [7,8,9,10,11,12]. Component measurements may show acceptable analytical validity under specific conditions [13,14,15,16], but that does not establish that a proprietary session score represents the physiological objective intended by a coach.

This distinction is important in competitive swimming. A session can contain warm-up, technical work, aerobic work, race-pace work, sprint elements, and recovery swimming. The session may therefore be mixed even when the coach has one predominant adaptive emphasis. In the Olbrecht framework, aerobic capacity (AEC), aerobic power (AEP), anaerobic power (ANP), and anaerobic capacity (ANC) describe intended emphases, not direct measurements of the metabolic or neuromuscular response [17,18,19].

WHOOP Activity Strain is a proprietary, algorithm-derived, nonlinear index. It is not a direct physiological measurement of metabolic load, neuromuscular stress, or training adaptation. To the authors’ knowledge, no published longitudinal single-athlete study has directly examined how WHOOP Activity Strain relates to exact coach-prescribed objective blocks, athlete-reported exertion, and completed swimming exposure across a competitive cycle in an elite junior swimmer.

The aim of this longitudinal single-case study was therefore to examine within-athlete associations among WHOOP Activity Strain, coach-prescribed physiological objectives, Session-RPE, and swimming exposure. A second aim was to determine whether the objective-comparison result was robust to different, transparently documented rules for resolving mixed and conflicting training-plan labels.

## 2. Materials and Methods

### 2.1. Study Design and Participant

This was an exploratory longitudinal single-case secondary analysis embedded in routine high-performance practice. Monitoring covered 4 January to 3 July 2026 (25.7 weeks). The analyzed data were generated through the athlete’s established training and performance-monitoring routine, independently of the research. Aquant training records, Session-RPE, and WHOOP monitoring were already used as part of normal coaching and athlete-support practice and continued unchanged throughout the monitoring period. No training session, test, intervention, or additional measurement was introduced specifically for this research. The participant was a 16.1-year-old male elite junior swimmer at the study entry. Before study entry, he had won a senior Hungarian national title and participated in the European Junior Championships. He specialized in 200–800 m freestyle. The findings and all inferential statements are restricted to this athlete.

The athlete completed 190 logged pool sessions totaling 1097.2 km and 364.25 h. The analysis did not include dry-land sessions. Short activation work performed around pool training was not treated as a separate analyzed session, and WHOOP activities not recorded as Swimming were not included in session-level analyses.

The exercise tests were conducted at the Exercise Physiology Laboratory of Széchenyi István University using equipment provided by Piston Ltd. (Budapest, Hungary). (European VAT number: HU 10465905) (26 September 2025). The test was performed on a treadmill following a protocol of gradually increasing intensity until complete exhaustion. The test protocol began with one minute of walking at a speed of 5 km/h, followed by running at 8 km/h. The speed was increased by 2 km/h every two minutes, with a continuous 2° incline. The athlete was instructed to run until exhaustion and received vigorous verbal encouragement throughout the test to achieve peak performance. During the test, peak oxygen uptake (VO_2_peak) was measured using equipment supplied by Piston Ltd. Resting heart rate (RHR) and maximum heart rate (HRmax) were also recorded using a chest-strap heart rate monitor. Participant and monitoring characteristics are summarized in Table 1.

### 2.2. Training Plans and Physiological Objectives

Training plans were created in the Aquant system as part of normal coaching. Training plans were created in the Aquant system as part of normal coaching. Aquant was an internally developed web-based planning system without a fixed commercial release version. Seven Long Distance PDF exports covering January–July 2026 were supplied for the revision. The PDFs were exported after the monitoring period, but the dated session content represented the archived training plans. Plan cards were matched to the athlete log by calendar date and AM/PM session. The displayed session distance and every explicitly labeled AEC, AEP, ANP, ANC, and Sprint block were extracted with source file and page retained for audit.

Sessions were not assumed to be physiologically pure. When a session contained more than one explicit AEC/AEP/ANP/ANC label, all labels were retained and the session was marked as mixed. For the single-category comparison, the predominant objective was defined as the explicit AEC, AEP, ANP, or ANC category accounting for the largest total prescribed swimming distance within the labeled objective blocks. All additional explicit objectives were retained as secondary objectives, and the session was flagged as mixed. A tie or the absence of an explicit AEC/AEP/ANP/ANC block resulted in no classification. Sprint was retained as session content but was not automatically converted into ANP or ANC. This operational rule was developed during the revision before the outcome models were rerun. It was not preregistered and should therefore be regarded as an exploratory classification rule. Classification was completed without consulting WHOOP Activity Strain or Session-RPE values.

This operational rule was developed during revision and was not preregistered. Analyses based on this rule were therefore post hoc and exploratory. Classification was completed without consulting WHOOP Activity Strain or Session-RPE.

If no Aquant plan card existed for a logged pool session, a unique pre-existing legacy coach label was retained. Five sessions met this fallback rule. The primary classification therefore prioritized the exact Aquant Long Distance plan. Sensitivity analyses used (i) the original legacy labels and (ii) a legacy-priority expanded classification that added Aquant labels only to previously blank sessions. This made the effect of the classification decision directly visible.

Sixteen sessions had different legacy and exact-plan labels. The populated coach-adjudication field was reviewed before the final analysis: all 16 decisions retained the exact Aquant predominant-objective label, so no manual override was introduced. The audit table records the legacy label, exact plan blocks, final decision, planned and completed distance, and the reason for each classification. The operational definitions of the four training objectives are summarized in Table 2.

### 2.3. WHOOP Monitoring and Data Extraction

The athlete wore a WHOOP 5.0 device (WHOOP Inc., Boston, MA, USA) on the wrist using the standard WHOOP wristband during the monitoring period. The current device screen showed firmware 50.41.1.0 after study completion; the historical firmware used on each study date could not be reconstructed and this version is not claimed for the entire period. The manufacturer does not publicly provide channel-specific PPG and accelerometer sampling frequencies for WHOOP 5.0, and the standard export did not contain raw sensor signals. These proprietary constraints limited technical reproducibility.

Data were obtained through the native WHOOP CSV export, not through an API. The workout file provided activity type, start and end time, duration, Activity Strain, energy expenditure, maximum and average heart rate, and time in heart-rate zones. Physiological-cycle and sleep files provided Recovery, HRV, algorithm-derived resting heart rate, and sleep duration. Respiratory rate and SpO2 were not analyzed and were removed from the analytical-variable list.

Session-specific Strain was the WHOOP Activity Strain exported for each matched Swimming workout. The score was not recalculated, rescaled, or summed from smaller fragments. Records were matched to the coach log using date, start time, AM/PM session identity, and session order. The strict primary match excluded entire multi-session dates when activity assignment remained ambiguous. WHOOP Activity Strain is described throughout as a proprietary algorithm-derived index, not a direct physiological measurement.

### 2.4. Competitions and Monitoring Scope

Four competition blocks occurred during monitoring: Muscat (30–31 January), the Hungarian National Championships in Sopron (15–19 April), the Hungarian Junior National Championships in Debrecen (3–6 June), and Gubbio (18–20 June). Across 14 competition dates, the raw WHOOP export contained 41 swimming activities. The export could not identify whether individual fragments represented warm-up, racing, or cool-down. Competition activities were not entered as coach-prescribed training sessions. Three logged Gubbio warm-up/cool-down sessions were explicitly excluded. The manuscript therefore does not describe monitoring as continuous throughout every competition or claim that the device was prohibited.

### 2.5. Session-RPE

The athlete reported a global modified CR-10 rating within 30 min after completion of each swimming session. Session-RPE Load was calculated as Session-RPE multiplied by logged session duration in minutes [20,21,22,23]. The within-30 min timing was a practical field adaptation of the approximately 30 min timing commonly used to reduce the influence of the final exercise bout.

### 2.6. Data Integration, Exclusions, and Missingness

The final log contained 190 pool sessions. Session-RPE was available for 188. Strict WHOOP Activity Strain matches were available for 171. Nineteen sessions were excluded from the strict WHOOP analyses: seven because activity fragments were ambiguous, four because no same-date swimming activity existed, three because fewer WHOOP activities than logged sessions were available, three competition warm-up/cool-down sessions, and two retained legacy matches on dates that were excluded in full under the strict rule. A device battery failure could not be demonstrated from the export and was not used as an invented explanation.

The primary objective was available for 134 sessions. Of these, 128 had a strict WHOOP match and 126 also had Session-RPE. The six classified sessions without strict WHOOP data were 9 March AM and PM, 6 April PM, 21 June AM, and 29 June AM and PM. Variable-specific complete cases were used.

Strict WHOOP exclusions were distributed across January (n = 2), February (n = 4), March (n = 3), April (n = 3), June (n = 6), and July (n = 1). Six excluded sessions had a final physiological objective: five AEC and one AEP. No ANP or ANC session was excluded because of missing strict WHOOP data. Missingness was therefore more frequent during the June post-competition/summer period but was not concentrated in the small ANP or ANC categories.

### 2.7. Statistical Analysis

The analysis was not preregistered, and no prospectively documented analytical plan existed. The final analysis was independently reproduced in Python 3.12.13 using statsmodels 0.15.0, pandas 3.0.5, NumPy 2.5.2, and SciPy 1.18.1. All inferential results reported in the manuscript were generated using the GEE implementation in statsmodels.genmod.generalized_estimating_equations with robust sandwich covariance.The statistical model was a Gaussian generalized estimating equation with an identity link. In practical terms, sessions from the same training week were treated as related rather than as fully independent observations. ISO week was used as the cluster because swimming was planned in weekly microcycles: sessions within a week shared training phase, load emphasis, and accumulated fatigue. Statsmodels Exchangeable was the primary within-week structure, and Statsmodels Autoregressive (grid = True) was used as the ordered within-week AR(1) sensitivity analysis. Omnibus Wald tests compared the four objectives. Pairwise contrasts were interpreted only after a significant omnibus test and were adjusted using Holm’s method.

Seven separate univariable GEE models related WHOOP Activity Strain to Session-RPE Load, Session-RPE, actual swimming distance, revised planned swimming distance, Recovery, HRV, and sleep duration. Robustness analyses repeated the objective comparison after excluding mixed-plan sessions, all 16 legacy-versus-plan conflicts, sessions completed more than 20% above or below prescribed distance, sessions without an exact Aquant card, and combinations of these restrictions. These thresholds were used only as transparent sensitivity checks; the primary analysis retained all strictly matched sessions with a final objective label. Throughout the manuscript, GEE coefficients are interpreted as session-level associations within this athlete, not as population-level effects. Two-sided *p* < 0.05 was used, but stability across analyses was given more weight than a single *p* value [24,25].

Because the dataset comprised repeated sessions from one athlete and only 24 weekly clusters, all inferential analyses were considered exploratory. In addition to the conventional robust covariance estimator, inference was examined using the Statsmodels bias-reduced covariance estimator as a conservative small-cluster check.

## 3. Results

### 3.1. Exposure and Data Availability

The athlete completed 190 logged pool sessions during the 25.7-week observation period. Strict WHOOP Activity Strain was available for 171 sessions, Session-RPE for 188, revised planned distance for 166, and the primary physiological objective for 134. Detailed descriptive distribution is provided in Table 3 without repeating all the summary values in the text.

### 3.2. Objective Classification from the Exact Plans

Aquant plan cards matched 161 of the 190 athlete sessions. An explicit AEC, AEP, ANP, or ANC block was identified in 129 sessions. Five additional sessions retained a unique legacy label because no Aquant card existed, giving 134 primary classifications. Seventeen athlete sessions contained more than one explicit manuscript objective. All 16 populated adjudications for legacy-versus-plan conflicts matched the exact-plan rule; consequently, the final coach-adjudicated classification introduced no manual overrides. The final distribution is shown in Table 4.

### 3.3. WHOOP Activity Strain Across Objectives

The conventional robust GEE suggested an overall difference in WHOOP Activity Strain across objectives (exchangeable *p* = 0.013; AR(1) *p* = 0.014). However, the difference was not statistically detectable using the bias-reduced covariance estimator applied as a conservative small-cluster check (exchangeable *p* = 0.080; AR(1) *p* = 0.075). The lower Activity Strain observed in ANP than in AEC should therefore be interpreted as a post hoc, exploratory within-athlete pattern rather than as a confirmed objective effect.

The result depended on the classification source. Repeating the strict model with the original legacy labels produced *p* = 0.542 (AR(1) *p* = 0.407). A legacy-priority expanded classification produced *p* = 0.259 (AR(1) *p* = 0.219). Thus, the exact-plan primary analysis suggested lower Activity Strain in ANP than AEC under the conventional covariance estimator, but this was not a classification-invariant finding.

Within the final exact-plan framework, the Statsmodels result was not driven by the disputed or mixed sessions. It remained after excluding mixed plans (n = 111; *p* = 0.004, AR(1) *p* = 0.007), all 16 legacy-plan conflicts (n = 112; *p* = 0.002, AR(1) *p* = 0.006), sessions completed outside 80–120% of prescribed distance (n = 117; *p* = 0.008, AR(1) *p* = 0.012), and sessions without an exact-plan card (n = 123; *p* = 0.011, AR(1) *p* = 0.008). The most restricted combination of non-mixed exact plans completed within 80–120% retained 97 sessions and also showed an objective difference (*p* = 0.002; AR(1) *p* = 0.002). These sensitivity analyses are summarized in Table 5.

Conventional robust-covariance *p* values are presented in the table. For the primary 128-session analysis, the corresponding bias-reduced *p* values were 0.080 for the exchangeable model and 0.075 for the AR(1) model.

### 3.4. Associations with WHOOP Activity Strain

WHOOP Activity Strain was positively associated with Session-RPE Load, Session-RPE, and actual swimming distance in both correlation structures. Revised planned distance showed a weak association in the exchangeable model (beta = 0.325, *p* = 0.040) but not AR(1) (*p* = 0.064), so this was not treated as a robust finding. Recovery, HRV, and sleep duration were not associated with Activity Strain. The corresponding session-level associations are reported in Table 6.

**Figure 1:**
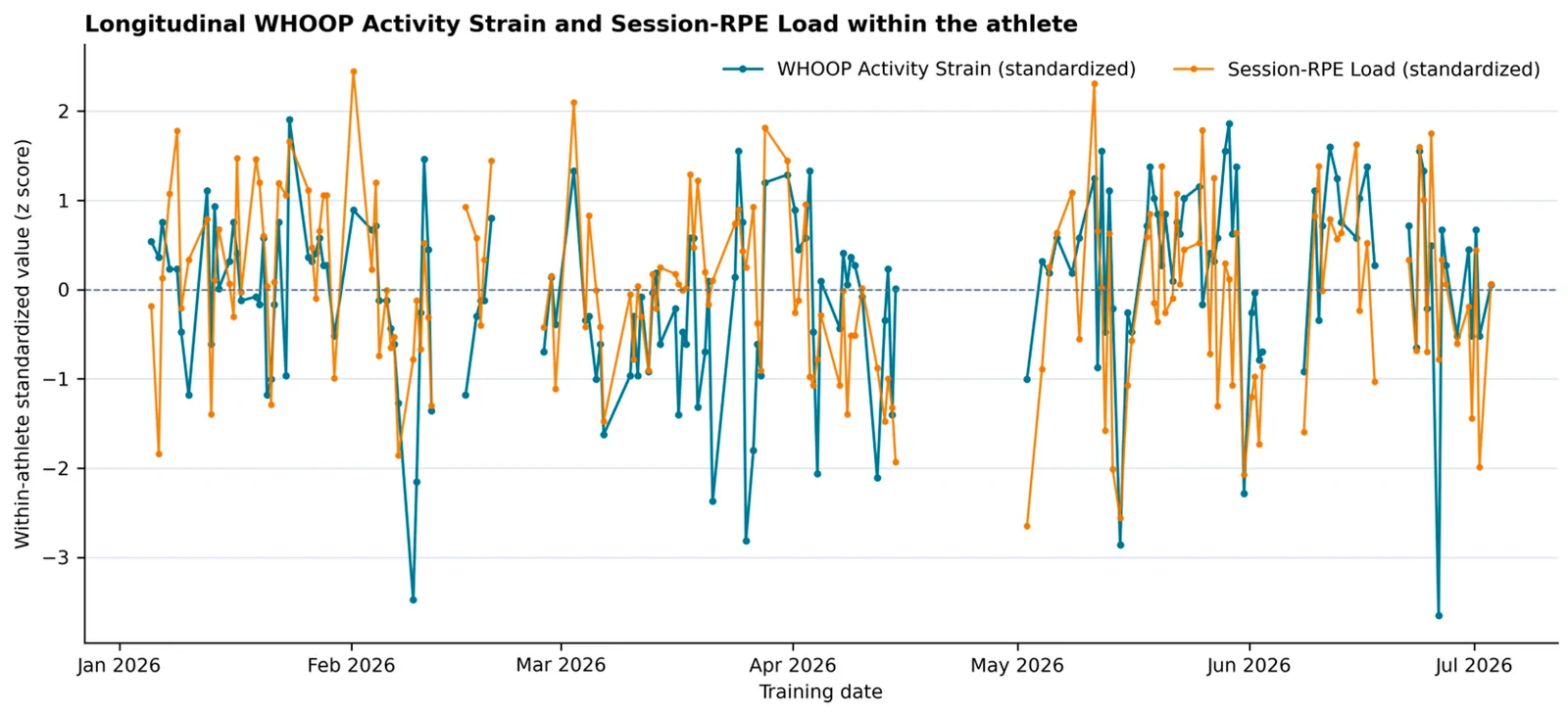
Longitudinal standardized WHOOP Activity Strain and Session-RPE Load for 169 strict matched sessions with both variables. Lines are broken across gaps longer than three days. The figure is descriptive; inference is based on the weekly clustered GEE model.

For 165 sessions with paired distances, mean planned distance was 5.930 km and mean actual distance was 5.946 km. The mean signed difference was +0.016 km per session and the total difference was +0.27%; however, the mean absolute session-level difference was 0.399 km. The small signed mean therefore reflected positive and negative deviations that largely canceled. The longitudinal variation in standardized WHOOP Activity Strain and Session-RPE Load is shown in Figure 1.

## 4. Discussion

### 4.1. Principal Findings

The post hoc exact-plan analysis suggested lower Activity Strain in ANP than AEC; however, the pattern was sensitive to both classification source and covariance estimator and should be treated as exploratory and hypothesis-generating. The associations of Activity Strain with Session-RPE, Session-RPE Load, and completed swimming distance were more consistent across analyses.

### 4.2. Why the Classification Rule Mattered

In these athletes’ training, the monitored swimming sessions were commonly mixed. A warm-up and aerobic volume can coexist with a short race-specific main set. Calling the entire session one category necessarily compresses information. The exact Aquant plans allowed all explicit objectives to be retained before selecting a predominant category. The older table combined plan variants and contained 16 conflicts with the exact Long Distance plan. All final adjudications retained the exact-plan label, and excluding all 16 conflicts strengthened rather than removed the omnibus result.

The exact-plan rule is auditable, but it is not a biological gold standard. The displayed Aquant block load is a planning value, and the largest block does not prove which physiological response dominated. Even so, the same overall finding survived removal of mixed plans and large delivery discrepancies. A stronger future design would still record the coach’s predominant goal, secondary goals, and main-set completion prospectively before each session.

### 4.3. Exploratory Interpretation of Lower ANP Activity Strain

In the post hoc exact-plan analysis, ANP sessions showed a lower mean Activity Strain than AEC sessions. A possible explanation is that brief high-intensity sets provide less time for a cumulative cardiovascular score to rise than long aerobic sessions. Short work may also involve substantial metabolic or neuromuscular demand that is not directly represented in a proprietary cardiovascular index [18,19]. These are hypotheses only: blood lactate, gas exchange, muscle oxygenation, and neuromuscular measures were not collected.

The ANP group contained only seven strict matched sessions, and ANC contained only five. The result should therefore be treated as a within-athlete signal requiring confirmation, not as a general device-validation result or a statement about elite junior swimmers as a population.

### 4.4. Perceived Load, Duration, and Completed Work

Session-RPE Load is Session-RPE multiplied by session duration. WHOOP Activity Strain is also sensitive to accumulated cardiovascular exposure over time. Their positive association may therefore be partly driven by a shared duration component and should not be interpreted as independent convergence of two duration-free constructs. The positive association with Session-RPE alone suggests that the relationship was not explained by duration only, but the two measures remain non-interchangeable [20,21,22,23,26].

Actual swimming distance was consistently associated with WHOOP Activity Strain. Revised planned distance was only weakly associated and lost conventional significance under AR(1). Planned and actual total distance were nearly equal, but the mean absolute session discrepancy was about 0.40 km. This supports using the completed session, not only the planned total, when interpreting wearable output.

### 4.5. Practical Applications

WHOOP Activity Strain provides an algorithm-derived indication of accumulated cardiovascular exposure during a session. It does not replace the written main set, the coach’s intended emphasis, the athlete’s RPE, or the completed distance. If those signals disagree, the disagreement should trigger review rather than automatic acceptance of one score.

Digital planning should store at least four fields: predominant objective, secondary objective(s), planned distance, and whether the main set was completed as written. That simple structure would reduce ambiguity and make future single-athlete monitoring more useful than relying on one retrospective category.

### 4.6. Strengths and Limitations

Strengths include dense session-level monitoring across 25.7 weeks, exact-plan exports, retention of mixed objectives, a documented 16-session coach adjudication, an auditable matching table, athlete-reported load, and robustness analyses that removed mixed, conflicting, poorly completed, and fallback-labeled sessions. All findings are explicitly restricted to one athlete.

Limitations include the single-case design; growth and maturation during adolescence; unbalanced objective counts; only seven ANP and five ANC sessions in the primary strict comparison; no direct metabolic or neuromuscular reference measure; and dependence of the result on the classification source. Aquant plan PDFs were exported after monitoring, although their dated contents represented archived plans. The WHOOP algorithm, raw sensor signals, sampling frequencies, and historical firmware were unavailable. Activity fragments on competition dates could not be separated into warm-up, racing, and cool-down from the export alone. Missingness and ambiguous multi-session matching reduced available observations. These limitations preclude causal or population-level inference.

The participant’s age (16.1 years) means that ongoing growth and biological maturation may have influenced physiological capacity and training responses during the monitoring period [27,28].

## 5. Conclusions

Within this athlete, WHOOP Activity Strain was consistently associated with perceived load and completed swimming distance. Objective-related differences arose from a post hoc classification rule, were sensitive to the classification source and covariance approach, and should therefore be considered exploratory and hypothesis-generating. WHOOP Activity Strain should be interpreted alongside the coach plan, secondary session goals, athlete feedback, and completed work. Confirmation in multiple athletes, using direct physiological reference measures and prospectively defined objective fields, is required.

## Figures and Tables

**Table 1:** Participant and monitoring characteristics.

Characteristic	Value
Sex	Male
Age at study entry	16.1 years
Height/body mass/BMI	187 cm/77.0 kg/22.0 kg·m^−2^
Competitive criterion	Senior Hungarian champion; European Junior Championships participant before study entry
Best World Aquatics score during the observation period	774 points in the 800 m freestyle on 31 January 2026
VO_2_ peak	72.2 mL·kg^−1^·min^−1^
Peak heart rate	197 beats·min^−1^
WHOOP-derived nightly resting HR	45.4 ± 1.8 beats·min−1 (n = 179 nights; range 41–53)
Observation period	4 January–3 July 2026 (25.7 weeks)
Pool sessions/distance/exposure	190/1097.2 km/364.25 h

**Table 2:** Operational definitions of the training categories.

Category	Intended Adaptive Emphasis Used in This Study
Recovery (REC)	Support recovery and restoration; retained in the coaching taxonomy but not present in the primary objective comparison.
Aerobic Capacity (AEC)	Increase sustainable aerobic work capacity.
Aerobic Power (AEP)	Increase high-rate aerobic energy delivery and race-relevant aerobic power.
Anaerobic Power (ANP)	Enhance the race-specific expression and rate of anaerobic energy supply during high-intensity efforts.
Anaerobic Capacity (ANC)	Develop the capacity to sustain and tolerate a high anaerobic contribution.
Sprint block	Retained as explicit plan content; not automatically forced into ANP or ANC.

Note. Categories describe intended adaptive emphasis; they are not proof of the physiological response produced in a session.

**Table 3:** Descriptive statistics of monitored variables.

Variable	n	Mean	SD	Median	IQR	Min	Max
WHOOP Activity Strain, strict matches	171	14.208	2.269	14.500	2.750	5.900	18.500
Session-RPE	188	5.963	1.338	6.000	2.000	1.000	9.000
Session-RPE Load	188	702.615	259.011	717.000	350.750	13.620	1341.000
Actual swimming distance (km)	189	5.805	1.260	5.900	1.500	2.000	9.000
Revised planned swimming distance (km)	166	5.933	1.166	6.100	1.500	3.000	8.900
Swimming duration (min)	190	115.026	27.038	119.000	34.500	2.000	167.000
WHOOP Recovery (%), strict matches	170	57.794	19.737	58.500	32.250	14.000	97.000
WHOOP HRV (ms), strict matches	170	42.876	11.365	43.000	16.000	15.000	77.000
WHOOP sleep duration (h), strict matches	170	7.428	0.938	7.275	0.979	5.417	10.750

**Table 4:** Distribution of the primary exact-plan classification.

Objective	Sessions	%	Planned km	% km	Strict WHOOP n
AEC	92	68.7%	568.1	69.7%	87
AEP	30	22.4%	183.4	22.5%	29
ANP	7	5.2%	35.5	4.4%	7
ANC	5	3.7%	27.5	3.4%	5

**Table 5:** Sensitivity analyses of the final coach-adjudicated objective comparison.

Objective	n	Exchangeable *p*	AR(1) *p*
Final labels—all strict	128	0.013	0.014
Exclude mixed-plan sessions	111	0.004	0.007
Exclude legacy-plan conflicts	112	0.002	0.006
Plan completion 80–120%	117	0.008	0.012
Exact Aquant plan card only	123	0.011	0.008
Pure exact plan + completion 80–120%	97	0.002	0.002

**Table 6:** Session-level associations with WHOOP Activity Strain within this athlete.

Session-Level Variable	n	Beta	95% CI	Primary *p*	AR(1) *p*
Session-RPE Load	169	0.00413	0.00316 to 0.00511	8.87 × 10^−17^	2.07 × 10^−14^
Session-RPE	169	0.74405	0.46788 to 1.02022	1.29 × 10^−7^	1.87 × 10^−7^
Actual swimming distance (km)	170	0.63413	0.27566 to 0.99260	0.000526	0.00225
Revised planned distance (km)	156	0.32494	0.01483 to 0.63505	0.04	0.0636
WHOOP Recovery (%)	170	−0.00811	−0.02944 to 0.01323	0.456	0.628
WHOOP HRV (ms)	170	−0.01310	−0.04550 to 0.01929	0.428	0.651
WHOOP sleep duration (h)	170	−0.23602	−0.55254 to 0.08049	0.144	0.131

## Data Availability

The datasets presented in this article are not publicly available due to privacy and ethical restrictions associated with the single-participant study design. Requests for further information should be directed to the corresponding author.
